# Applicability of the Rayleigh equation for enantioselective metabolism of chiral xenobiotics by microsomes, hepatocytes and *in-vivo* retention in rabbit tissues

**DOI:** 10.1038/srep23715

**Published:** 2016-03-29

**Authors:** Shifra Jammer, Faina Gelman, Ovadia Lev

**Affiliations:** 1 The Casali Center of Applied Chemistry, The Institute of Chemistry, The Hebrew University of Jerusalem, Jerusalem 91904, Israel; 2 The Geological Survey of Israel, Jerusalem 95501, Israel

## Abstract

In this study we propose a new approach for analyzing the enantioselective biodegradation of some antidepressant drugs mediated by human and rat liver microsomes by using the Rayleigh equation to describe the enantiomeric enrichment−conversion dependencies. Analysis of reported degradation data of additional six pesticides, an alpha blocker and a flame retardant by microsomes or hepatocytes *in vitro* reaffirmed the universality of the approach. In all the *in vitro* studied cases that involved enantioselective degradation, a Rayleigh dependence of the enantiomeric enrichment was observed. Published data regarding *in vivo* retention of myclobutanil in liver, kidney, muscle and brain tissues of rabbits following injection of the racemate were remodeled showing prevalence of the Rayleigh law for the chiral enrichment of the fungicide in the various tissues. This approach will revolutionize data organization in metabolic pathway research of target xenobiotics by either liver microsomes, hepatocytes or their organ-specific *in vivo* retention. The fact that the enantiomeric enrichment as a function of the conversion can be described by a single quantifier, will pave the road for the use of structure activity predictors of the enantiomeric enrichment and for mechanistic discrimination based on parametric dependence of the quantifier.

Enantioselective biodegradation analysis of pharmaceuticals, pesticides and other substances of environmental and toxicological interest has been extensively researched over the past decade due to the different physiological effect of the different enantiomers^1^,^2^,^3^. The biotransformation and detoxification of most xenobiotics takes place in the liver and is often enantiomer dependent^4^. Several *in vitro* liver models have been developed for metabolism research and toxicity assessment in the past few decades, including microsomes, cytosol, cell lines, primary hepatocytes etc., while with each model having its own advantages and disadvantages^5^. Liver microsomes contain the class of cytochrome P450 enzymes which are the most important mammalian detoxifiers, responsible for the first degradation step (phase I oxidation) of hydrophobic drugs^6^. Therefore, liver microsomes are generally used for drug biotransformation and metabolic profiling, when the predominant route of metabolism is known to be phase I oxidation by microsomal pathways. Hepatocytes, in contrast to liver microsomes, also contain phase II enzymes. Therefore they can provide valuable information which will complement liver microsomal data^7^,^8^ and are often used in drug toxicity research due to their strong resemblance of *in vivo* activity in livers^5^,^9^.

*In vitro* and some *in vivo* enantioselective investigations are commonly interpreted by three main methods. (І) The first method examines the enantioselective degradation of racemic compounds by determining the ratio between the enantiomers at a termination point of the reaction as the enantiomeric excess (ee)^10^, enantiomer fraction (EF)^11^ or enantiomeric ratio (ER)^11^ (i.e (R − S)/(R + S), R/(R + S) and R/S, respectively, where R and S denote concentrations of the two enantiomers)^12^,^13^,^14^,^15^,^16^. These parameters are conversion dependent, and thus the enrichment obtained at one test provides only little meaningful information for prediction of the enrichment obtained at other reaction end points. (II) The second method examines the enantioselective metabolism of racemic compounds. Concentration - time profiles of the individual enantiomers are constructed providing the rate coefficients of the individual enantiomers as well as time dependent EF values^17^,^18^,^19^,^20^,^21^,^22^,^23^,^24^,^25^,^26^. The characterization of the enantiomeric enrichment requires a large number of constants and therefore complicates comparative analysis. (Ш) The third approach operates with enzyme kinetic analysis which provides the K_m_ and V_max_ Michaelis Menten kinetic constants for the individual enantiomers^27^,^28^,^29^,^30^,^31^,^32^,^33^,^34^,^35^,^36^,^37^,^38^,^39^,^40^,^41^. This approach forces the use of pure enantiomer standard solutions in order to examine the influencing and interfering effects arising from the chiral recognition of the enzymes binding site. The use of pure enantiomer standard solutions is expensive and limited since these standards are not always available.

In this manuscript we propose a different method to simplify and generalize the information provided by methods I and II for analyzing the enantioselective biodegradation which is advantageous over method І and can simplify method II. The method suggests the use of the Rayleigh equation to describe the enantiomeric enrichment - conversion relationship in *in vitro* and *in vivo* systems. It was recently demonstrated that under relevant environmental conditions the Rayleigh equation, usually used in compound specific isotope analysis (CSIA)^42^, is also effective in describing the enantioselective behavior. The proportionality constant called the enantiomeric enrichment factor, ε_ER_ (equation 1) is not conversion dependent and therefore can be used as an identifying tool for a specific reaction^43^,^44^,^45^,^46^ and can be predicted by QSAR analysis^47^.


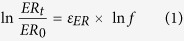


*ER*_t_ and *ER*_0_ represent the initial and conversion-dependent enantiomeric enrichments (ER = [R]/[S]) and *f* is the residual fraction (*C*_t_/*C*_0_).

In a previous article^43^ we have defined the enantiomeric enrichment factor in equation 1 by equation 2.


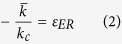


where, *k*_c_ is the observed overall first order rate constant of both enantiomers, and 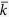 is the difference between the individual first order rate constants of each enantiomer (*k*_1_ − *k*_2_). Alternative and at least equally applicable forms of the Rayleigh equation^48^,^49^, connected by the relationship^43^: 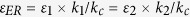, are defined in the Supplementary online. The use of the Rayleigh equation can simplify the way of presenting the enantioselective degradation by expressing the reaction kinetics (overall and individual coefficients) and the enantiomeric enrichments (EF) - in one constant. Furthermore, as demonstrated in a previous article^47^, the enantiomeric enrichment factor for isolated enzymatic reactions can be predicted by QSAR analysis and hence may be useful in the QSAR of *in vitro* or *in vivo* biodegradation reactions that consist of multiple enzyme activities.

The present study investigates *in vitro* the implementation of the Rayleigh equation during enantioselective degradation of several antidepressant drugs (Fig. 1) by liver microsomes from humans and several other rat species (Table 1). The fact that a number of enzymes of varying activities are involved in the metabolic pathway may complicate the observed kinetics and raise doubts regarding the applicability of the Rayleigh enrichment equation for the description of liver detoxification transformations. In order to generalize our findings, we also examined a set of articles that were reported between 2011 and 2015 in which sufficient data was provided for *in vivo* or *in vitro* microsome or hepatocyte mediated transformations to calculate the individual reaction kinetics of the two enantiomers and to assess their enantiomeric enrichments.

## Results and Discussion

### Kinetic degradation in liver microsomes

**Figure 2:**
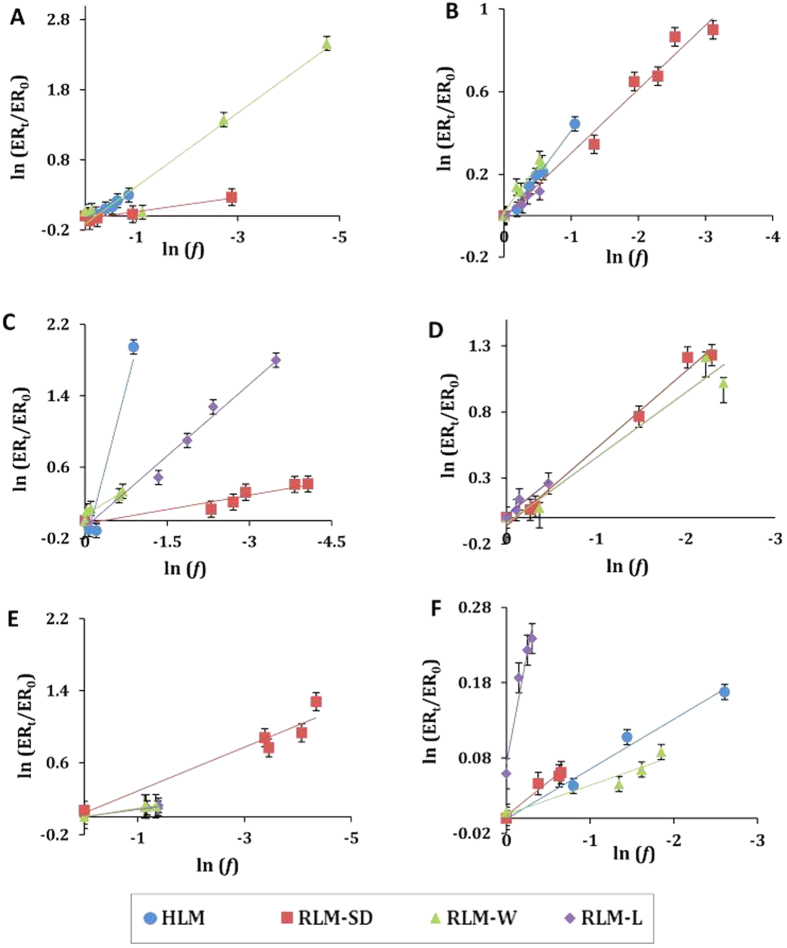
Rayleigh plots for the enantioselective biodegradation of antidepressant drugs using liver microsomes. HLM- human liver microsomes; RLM- rat liver microsomes; SD- *Sprague Dawley*, W- *Wistar Han* and L- *Lewis*. (**A)** CTM; (**B)** MTZ; (**C)** MNS**; (D)** FLX**; (E)** N-FLX**; (F)** O-DMV. Data are expressed as the mean ±  standard error of the mean, n = 3.

The kinetic tracking of the de-methylation of *(R,S*)-fluoxetine (FLX), (*R,S*)-norfluoxetine (N-FLX), (*R,S*)-citalopram (CTM), (*R,S*)-venlafaxine (VNF), (*R,S*)-O-desmethylvanlafaxine (O-DMV), (*R,S*)-mirtazapine (MTZ), and (*R,S*)-mianserin (MNS) gave a first order kinetic fit with overall rate constants (*k*_c_) in the range of 0.036–0.14 hr^−1^ (Table 2 and Supplementary Fig. S1 online) and correlation coefficients in the range of 0.9–0.95. The individual first order rate constants of each enantiomer (*k*_1_, *k*_2_) are detailed in Table 3. The linear fit to the Rayleigh approximation was obtained for the enantiomeric enrichment (Fig. 2, R^2^ = 0.92–0.99) obtaining the enantiomeric enrichment factors detailed in Table 4.

It is clearly observed from Fig. 2 and Table 4, that there is a wide dispersion of the enrichment factors between the different microsome sources. The enantiomeric enrichment factors vary from high (i.e ε_ER_ = −237 ± 29 for MNS degraded by human liver microsomes) to very low values (i.e ε_ER_ = −4 ± 1 for O-DMV degraded by *Wistar Han* rat liver microsomes) and in some cases remained close to racemic composition (NE), with very small increase in the enrichment ratio of up to 9% (i.e. the ER for VNF was 1.1 ± 0.08 through all the incubations with the various microsomes).

Although there is an overlap between the different CYP’s in the various microsomes (Table 1) we are unable at this stage to use the enrichment factors for characterizing the specific CYP enzyme that determines the enantioselective degradation. Despite the fact that the same CYP families are responsible for the degradation of the different substances, there is a high variability in the enantiomeric enrichment factors and the order of enrichment (ranking) is very different between them. For example, HLM exhibited the highest enrichment parameter (and thus selectivity) compared to all the investigated microsomes for N-FLX but also the lowest ε_ER_ for CTM and FLX. CTM exhibited the highest ε_ER_ among all the tested drugs (by RLM-W) but it also exhibited the lowest ε_ER_ in this study (by RLM-L and HLM).

### Reconstruction of the Rayleigh dependence based on reported biodegradation studies

In addition to the microsome studies, the Rayleigh equation was fitted to reported studies on biodegradation of chiral compounds using liver microsomes and hepatocytes for which there was sufficient data available. We focused on studies published on years 2011–2015 that reported on first order kinetics and/or provided concentration - time profiles of the individual enantiomers after incubation of the racemate with the metabolizer, i.e. systems analyzed by approach II in the introduction. From reconstruction of this data, one can derive the Rayleigh dependence and provide the enantiomeric enrichment factor.

### Enantioselective biodegradation using liver microsomes

1. Wu. *et al*.^19^ reported on the enantioselective degradation of the fungicide (2RS, 3RS)-paclobutrazol in rat liver microsomes. The authors provided the individual first order degradation rate constants as 0.0373 min^−1^ and 0.0634 min^−1^ for (2R, 3R)-paclobutraz and (2S, 3S)-paclobutrazol, respectively. Reconstruction of the Rayleigh dependence, based on the data provided in Fig. 3A in ref. 19 obtained the enantiomeric enrichment factor of −66 ± 14% with a correlation coefficient of 0.97.
2. Shen *et al*.^20^ reported on the metabolism of the systemic fungicide hexaconazole and its enantiomers in rat liver microsomes. The authors provided the individual first order degradation rate constants for the metabolism with male Sprague–Dawley rat liver micrsomes as 0.09324 min^−1^ for (+)-hexaconazole and 0.06804 min^−1^ for (−)-hexaconazole. Reconstruction of the Rayleigh dependence, based on the data provided in Fig. 3A in ref. 20 obtained the enantiomeric enrichment factor of −37 ± 2% (R^2^ = 0.99).
3. Zhang *et al*.^22^ reported on the stereoselective degradation of the metalaxyl (MX), a phenylamide fungicide, in rat and rabbit hepatic microsomes. Based on the data in Fig. 3a in ref. 22 providing the concentration of each enantiomer during the time of the degradation experiment, we have calculated the individual degradation constants for (+)-S-MX and (−)-R-MX to be 0.0864 and 0.0415 min^−1^ (R^2^ = 0.98), respectively (these values were reinsured with the half-lives provided by the authors as 7.71 min and 15.58 min for (+)-S-MX and (−)-R-MX, respectively). Based on the same data as above we were able to reconstruct the Rayleigh dependence with ε_ER_ = −78 ± 14% (R^2^ = 0.94).
4. Ma *et al*.^50^ reported on the enantioselective metabolism of the chiral herbicide diclofop-methyl (DM) in fish liver microsomes (loach). The authors provided the individual first order degradation rate constants as 0.0346 and 0.1904 min^−1^ for (R)-DM and (S)-DM, respectively. Reconstruction of the Rayleigh dependence, based on the data provided in Fig. 3A in ref. 50 obtained the enantiomeric enrichment factor of −142 ± 40% (R^2^ = 0.94).
5. Esslinger *et al*.^21^ investigated the degradation of the enantiomers of α-, β-, and γ-hexabromocyclododecane (HBCD), brominated flame retardant, by using induced rat liver microsomes. Based on the data provided in Fig. 1 in ref. 21 on the degradation of the enantiomers of γ-HBCD we calculated the enantiomeric enrichment factor to be −89 ± 13% (R^2^ = 0.98) and the authors provided the individual first order rate constants as 0.021 and 0.060 min^−1^ for (+)-γ-HBCD and (−)-γ-HBCD, respectively.

### Enantioselective biodegradation using hepatocytes

As aforementioned in the introduction, liver microsomes are the most popular *in vitro* model in drug biotransformation research. However they contain only phase I metabolizing enzymes and results obtained with microsomes cannot be used for quantitative estimations of *in vivo* biotransformation^5^, because CYPs and UGTs are enriched in the microsomal fraction and there is no competition with other phase II enzymes. Therefore hepatocytes are envisaged as a better model system because of the full complement of both phase I and phase II metabolizing enzymes, along with the presence of transporter proteins, which should result in drug concentrations within the hepatocyte that are equivalent to *in vivo* concentrations within the liver^9^. The ability to fit the Rayleigh equation to reported studies on biodegradation of chiral compounds using hepatocytes is important for investigating the implementation of using the Rayleigh equation in a more accurate *in vitro* model.

Wang *et al*.^24^ studied the enantioselective metabolism of the fungicide hexaconazole as well, they used male Sprague–Dawley rat hepatocytes. In this case the reported individual first order rate constants are 0.036 and 0.134 for (+)-hexaconazole and (−)-hexaconazole, respectively. Reconstruction of the Rayleigh dependence based on the time dependent metabolism shown in Fig. 3a in ref. 24, obtained the enantiomeric enrichment factor of −184 ± 6% (R^2^ = 0.99). The significant difference in the enantiomeric enrichment factors (−37 ± 2% in Shen *et al*.^20^ and −184 ± 6% in Wang *et al*.^24^) can result from the use hepatocytes over microsomes^51^. In a second test case, Wang *et al*.^52^ studied the degradation of the fungicide myclobutanil in hepatocytes from male Sprague–Dawley rats. The authors provided the individual first order rate constants as 0.065 h^−1^ for (+)-myclobutanil and 0.046 h^−1^ for (−)-myclobutanil. Reconstruction of the Rayleigh dependence, based on the data provided in Fig. 3a in ref. 52 obtained the enantiomeric enrichment factor of −24 ± 2% (R^2^ = 0.97). Two more test cases are reported by Xu *et al*. studying the stereoselective metabolism of the chiral herbicides ethofumesate (ETO)^53^ and fluroxypyr methylheptyl ester (FPMH)^54^ using hepatocytes from male Sprague-Dawley rats. In both cases the author provided the first order half-life values as 2.0 h and 4.1 h and 5.1 min and 13.1 min for (+) and (−) ETO and FPMH, respectively. Reconstruction of the Rayleigh dependence based on the concentration–time curves shown in Fig. 2 in ref. 53 and in Fig. 4 (for 10 μM) in ref. 54 obtained the enantiomeric enrichment factor of −75 ± 11% (R^2^ = 0.96) and −120 ± 30% (R^2^ = 0.96) for ETO and FPMH, respectively.

### Enantioselective biodegradation using *in vivo* methods

Surprisingly, we could demonstrate that the applicability of the Rayleigh equation is not limited to *in vitro* studies but rather to *in vivo* studies as well, as long as concentration - time profiles are available and they obey logarithmic decay or accumulation over time. For example, Sun *et al*.^26^ have reviewed the enantioselective behavior of the fungicide myclobutanil enantiomers in variant rabbit tissues following intravenous administration of the racemic fungicide. Using the plasma concentration-time curves of the two enantiomers (presented in Fig. 1A in ref. 26) we obtained the enantiomeric enrichment factor of −28 ± 5 (R^2^ = 0.97). Analysis of the distribution of myclobutanil in various tissues, presented in Fig. 3 in ref. 26, obtained the enantiomeric enrichment factors of −33 ± 16 (R^2^ = 0.92) for the liver, −20 ± 6 (R^2^ = 0.97) for the brain, −32 ± 16 (R^2^ = 0.89) for the kidney and −21 ± 6 (R^2^ = 0.97) for the muscle. Notably, in all these cases the enantiomers decay followed first order dependence over the corresponding time frame, which is a prerequisite for obtaining Rayleigh dependence. However, in rabbits’ hearts and fats the decay of the myclobutanil does not follow linear kinetics and in these cases the enrichment does not adhere to the Rayleigh law. The fungicide decay in the various organs of rabbits allow us to demonstrate how the Rayleigh presentation allows rapid comparison between and ranking of the enantio-specific decay rate of myclobutanil in different organs (Liver and kidney > muscle and brain ≫ lung). This quantitative ranking is obscured by the large number of time trace data points in the original presentation. Another example can be presented by the study of Kong *et al*.^25^ reporting on the stereoselective metabolism of the alpha blocker doxazosin (DOX) *in vivo* in male Sprague–Dawley rats. Using the data in Fig. 3 in ref. 25 representing the plasma concentration-time profiles of (−)-DOX and (+)-DOX we could demonstrate prevalence of the Rayleigh law giving an enantiomeric enrichment factor of −45 ± 13 (R^2^ = 0.92).

The results of the present study indicate that the use of the Rayleigh equation can simplify the data and can serve as a tool for comparison and prediction. As it was already mentioned in the introduction, the enantioselective biodegradation in *in vitro* and *in vivo* systems is usually interpreted by three main methods: (I) Determination of the ratio between the enantiomers at a termination point of the reaction; (II) Determination of the rate coefficients of the individual enantiomers as well as time dependent EF’s; (III) determination of the Michaelis Menten kinetic constants for the individual enantiomers. The use of the Rayleigh equation to describe the enantiomeric enrichment - conversion relationship is advantageous of method І and can simplify and generalize the information provided by methods II and III as following:

The enrichment parameters used in method І (EE,ER and EF) are conversion dependent, therefore enrichment values obtained at one test provides only little meaningful information for prediction of the enrichment values that will be obtained at other reaction end points. In contrast, the enantiomeric enrichment factor (ε_ER_) is not conversion dependent and therefore can be used as an identifying tool for a specific reaction.

Method II uses four constants (overall and individual first order rate coefficients as well as EF values) in order to characterize the enantioselective degradation. Whereas the Rayleigh equation provides the enantiomeric enrichment factor that can characterize the relative degradation rates as described in equation 2, expressing the reaction kinetics (overall and individual coefficients) and the enantiomeric enrichments (EF) - in one constant. The use of a large number of constants complicates comparative analysis; hence the use of the Rayleigh equation can simplify the data and can serve as a tool for comparison and prediction.

In a previous article^43^ we have demonstrated that the use of the Rayleigh equation is restricted to cases where the concentration of the substrate is much lower than the saturation constant, K_M_, and linear overall degradation kinetic process is observed. Thus, the use of racemic solutions is adequate for this method as in these concentrations the interactions influencing the K_M_ are negligible. Therefore, the use of the Rayleigh equation is affordable over method Ш that requires the expensive and limited use of pure enantiomer standard solutions in order to determine the individual saturation constants.

### Enantiomeric analysis and CSIA

The process of comparing the enrichment of one species relative to the other to prove the existence of biodegradation can be applied in both compound specific isotope analysis (CSIA) and enantiomeric analysis^43^,^55^. In CSIA, transformation mechanisms and associated degradation pathways can be inferred because the isotope fractionation reflects the different transition states^56^. Therefore isotope fractionation analyses (especially dual isotope analysis) may potentially provide information about the strategy of microorganisms to break down contaminants at the enzymatic level^56^, identifying the responsible enzyme for degradation in multiple enzyme environment. However, it is noteworthy that in some cases the isotope effect of a degradation process is obscured by other non-biological factors involved in the process such as diffusion through the membrane^57^, and that carbon CSIA has a general limitation to be suitable mostly for only relatively small (<C_12_) organic molecules, due to “dilution effect”^58^ (i.e. only small fraction of the isotopes in a large molecule participate in a particularly bond cleavage). This upper limit for the size of compounds that can realistically be investigated by CSIA, eliminates large compounds as pharmaceuticals and other common micropollutants. In contrast, enantiomeric analysis has the ability to analyze large compounds. Currently, it is limited to giving information regarding the conjugation and accessibility for overcoming different degradation processes. In most cases it cannot explicate the mechanistic bond cleavage pathway, unless it is measured in combination with isotope analysis (ESIA)^55^. Although we are now unable to use the enantiomeric enrichment factors for characterizing the specific enzyme that determines the enantioselective degradation in the microsomes studies mentioned above, once a sufficient data base of enantiomeric enrichment factors will be available, it will enable construction of QSAR dependencies for families of compounds. The fit of a particular QSAR of a family of compounds will allow identifying the enzyme responsible for the degradation in biodegradation reactions, *in vivo* and *in vitro*, that consist of multiple enzyme activities.

### The Eudismic ratio and Rayleigh equation

In pharmacodynamics and toxicology practitioners refer to the eudismic ratio, EuR, defined as ratio of the bioactivity, BA (per gram), of the active enantiomer (the eutomer) to that of the less active enantiomer (the distomer)^59^. The eudismic ratio is often expressed as the ratio between the mortality or inhibitory inducing doses, i.e. EC_50_ or IC_50_ of the two enantiomers (EC_50_ and IC_50_ being the mortality or inhibitory doses affecting half of the exposed population). The eudismic ratio is therefore organ- or population specific, but it is independent of the concentration or dose of the two enantiomers. Assuming linear dose - response relationship^60^, then one can express equation 1 in terms of the initial and residual bioactivity of the two enantiomers BA_i,t_, where, i is the enantiomer index (1 or 2). BA_i,t_ can be expressed as the product of the relevant concentration of the drug, the relevant (ingested) volume and the specific biological activity of the drug, and then equation 1 is readily transformed into equation 3


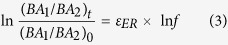


Thus, although the bioactivity ratio of the two enantiomers, (BA_1_/BA_2_)_t_ depends on the eudismic ratio, (e.g., (BA_1_/BA_2_)_t_ = ([R]/[S])·EuR), the same term appears in the denominator and numerator of the left hand side of equation 3 and cancels off. Thus, the Rayleigh equation is independent of the eudismic ratio; at least as long as linear dose response is retained.

## Conclusions

The conclusion arising from our microsomes biodegradation of antidepressant drugs and the reconstruction of the Rayleigh dependence in the reported studies said above is clear: As long as first order kinetics takes place, the Rayleigh equation is suitable for describing the enantioselective biodegradation *in vivo* and *in vitro* and not only in isolated enzyme reactions. This was assessed on three levels: (i) *In vitro* microsome studies (our own, as well as reported) of various drugs, pesticides and fungicides which follow the Rayleigh law despite having a consortia of cytochrome P450 rather than a single active enzyme, (ii) *In vitro* rat hepatocytes studies, which similar to microsomes, have several phase I enzymes and in addition have phase II conjugation activity, that also obeyed the Rayleigh law in all four test cases examined. And finally, (iii) *In vivo* studies where the Rayleigh equation is helpful in describing the complex transport/decay processes, at least when the individual transport or decay process obeys logarithmic dependence on time. The research thus paves the road for the use of structure activity relationship for the prediction of chiral metabolism in the environment as well as retention of xenobiotics in various mammalian tissue cells.

## Methods

### Materials and reagents

(*R,S*)-mianserin (99%), (*R,S*)-venlafaxine (99%) and *(R,S*)-fluoxetine (99%) were supplied by Sigma Co. Ltd. 0.5 M potassium phosphate buffer pH 7.4 (0.2 μM filtered), NADPH regenerating system solutions A (containing: 26 mM NADP+, 66 mM glucose-6-phosphate, 66 mM MgCl_2_ in H_2_O) and B (containing: 40U/Ml glucose-6-phosphate dehydrogenase in 5 mM sodium citrate), pooled male rat liver microsomes (*Sprague Dawley, Wistar Han*), human liver microsomes-50 donor pool (20 mg protein/mL) and insect control supersomes (5 mg protein/mL) were supplied by BD Gentest. (*R,S*)-mirtazapine (99%) was supplied by Zotal Ltd. (*R,S*)-norfluoxetine (99%), (*R,S*)-citalopram (99%), (*R,S*)-O-desmethylvanlafaxine (99%) and Verapamil (98%) was supplied by Tzamal D-Chem Laboratories Ltd. Female rat liver microsomes (*Lewis*) was obtained from the MicroLiver Technologies Laboratory, the Hebrew University of Jerusalem. All organic solvents were HPLC grade and were supplied by Bio-Lab Ltd.

Liver microsomes were stored at −80 °C and thawed rapidly in 37 °C prior to use. Stock solutions were prepared in methanol at 1 mg/mL and stored in −20 °C before use. Working standard solutions were prepared by diluting the stock solutions with the proper reaction solvent.

### *In vitro* biotransformation experiments

The kinetic tracking of the de-methylation of: FLX, N-FLX, CTM VNF, ODV, MTZ and MNS was conducted in parallel separate 1.7 mL micro-centrifuge tubes, the whole content of each tube was used for a single analysis. Incubations were performed for 0–120 min at 37 °C in a shaking water bath. The reactions were initiated by the addition of 120 μl NADPH-regenerating system (100 μL of solution A and 20 μL of solution B) that were added after a 3-min pre-incubation of the incubation mixtures containing 10 μl of substrate (1 g/L), 50 μl human/rat hepatic microsomes (20 mg protein/ml) and 270 μl sodium phosphate buffer, 0.1 M pH 7.4 final volume was 500 μl. The reactions were stopped at time intervals by cooling on ice and addition of 100 μl acetonitrile followed by the addition of 20 μL internal standards stock solution (0.1 g/L). After termination of the reaction 200 microliter sodium carbonate solution (1 M, pH 12) was added and samples were then vigorously shaken for 1 min and consequently extracted into 6 ml n-hexane/acetonitrile (98/2 vol/vol) using an Intelli mixer on 50 rpm for 5 min. after centrifugation for 10 min (6,000 rpm) the upper organic layer was collected and the procedure was repeated. The samples were eventually evaporated till dry under a gentle stream of nitrogen. The residue was dissolved in 200 microliter methanol and analyzed by HPLC-MS. These degradation studies were done in duplicates.

Control assays were run under the same conditions, with 200 μL insect control supersomes (5 mg protein/ml) used as a negative control, i.e containing no CYP450 but the same protein content (cytochrome b5), while 10 μl of Verapamil (1 g/L) and 50 μl human microsomes (20 mg protein/ml) was incubated for 30 min as a positive control.

Recoveries of the analyzed drugs were 89 ± 9% (VNF), 88 ± 19% (ODV), 95 ± 12% (MNS), 95 ± 6% (MTZ), 98 ± 4% (CTM) 96 ± 3% (FLX) and 92 ± 15% (N-FLX), similar to a previous report using the same extraction method for FLX and N-FLX^61^. The recovery data is reported as average number  ±  standard deviation (n = 3).

### Chromatographic and analytical conditions

Electrospray ionization mass spectrometry (ESI–MS) analysis was conducted using an Agilent 6520 high accuracy Quadrupole Time of Flight (QTOF) mass spectrometer, equipped with a chiral column (CHIROBIOTIC V, 25 cm × 4.6 mm × 5  μm; Astec). The chiral separation method was based on methodology of Begnall *et al*.^62^. Separation of VNF, ODV, FLX and CTM was undertaken using isocratic conditions, with a mobile phase of: methanol containing 4 mM ammonium acetate and 0.005% formic acid at a flow rate of 0.8 ml/min; for the separation of MNS and MTZ the same chromatographic condition were uses except for the flow rate of 0.9 ml/min and the separation of N-FLX was undertaken using isocratic conditions, with a mobile phase of: methanol containing 4 mM ammonium acetate and 0.005% formic acid:water (90:10) at a flow rate of 0.8 ml/min. For all separations the column and autosampler were maintained at 25 °C, with an optimal chromatographic run time of 24 minutes, the injection volume was 10 μL. QTOF mass spectrometer equipped with an electrospray ionization source (Agilent G3251A Dual ESI) was used for chiral drug identification and quantification. Analysis was performed in positive mode with a capillary voltage potential of 4000 V; the nebulizer gas pressure was set to 40 psi and drying gas flow was 10 L/min, with a drying gas temperature of 300 °C. The fragmentor voltage was set at 140 V, and skimmer voltage was 65 V. Scan range was 50–1000 m/z, the other MS parameters remained at auto-tune conditions.

The enantiomeric ratios were expressed as the ER – the peak area of one enantiomer (the more abundant) divided by that of the other enantiomer.

## Additional Information

**How to cite this article**: Jammer, S. *et al*. Applicability of the Rayleigh equation for enantioselective metabolism of chiral xenobiotics by microsomes, hepatocytes and *in-vivo* retention in rabbit tissues. *Sci. Rep*. **6**, 23715; doi: 10.1038/srep23715 (2016).

## Supplementary Material

Supplementary Information

## Figures and Tables

**Figure 1:**
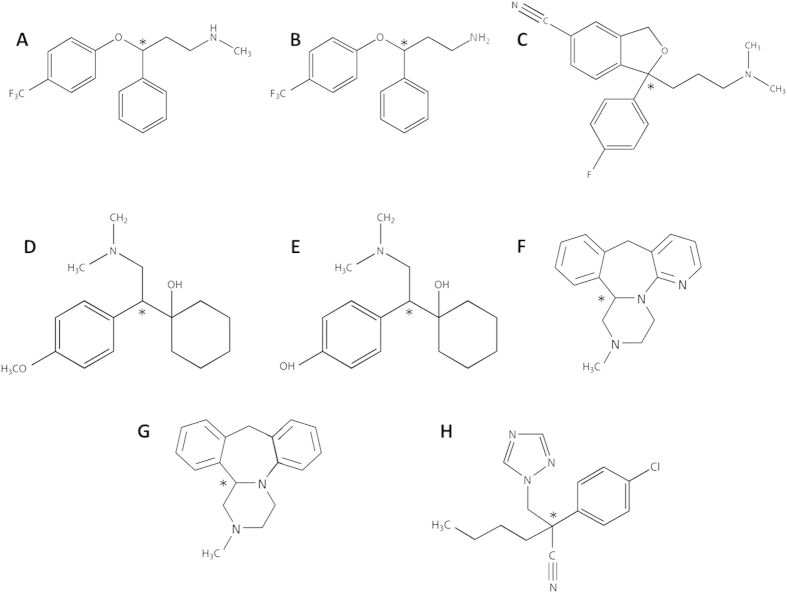
Chemical structures of the chiral antidepressant drugs. (**A–C)** Selective serotonin reuptake inhibitors (SSRIs): (R,S)-fluoxetine (FLX), its main metabolite (R,S)-norfluoxetine (N-FLX) and (R,S)-citalopram (CTM); (**D,E)** Serotonin– norepinephrine reuptake inhibitors (SNIR) :(R,S)-venlafaxine (VNF) and its main metabolite (R,S)-O-desmethylvanlafaxine (ODV); (**F,G)** Tetracyclic antidepressants TTA: (R,S)-mirtazapine (MTZ) and (R,S)-mianserin (MNS); (**H**) myclobutanil, a conazole class fungicide. Chiral center is denoted by an asterisk (*).

**Table 1:** Characteristics of the different liver microsomes used in this research.

Microsomes source	Enzyme measured	Enzyme activity [pmol/(mg × min)]
*Wistar Han* rat liver microsomes (RLM-W)	CYP 3A	3000α
	CYP 1A	150
	CYP 2C	3300
*Sprague Dawley* rat liver microsomes (RLM-SD)	CYP 3A	4900α
	CYP 1A	290
	CYP 2C	3200
*Lewis* rat liver microsomes (RLM-L)	CYP 1A1/1A2	301β
Human liver microsomes (HLM)	CYP 3A4	86α
	CYP 1A2	540
	CYP 2C9	2900
	CYP 2D6	81

^α^Information provided by BD Gentest (pooled male *Wistar Han* rat liver microsomes, pool of 54; pooled male *Sprague Dawley* rat liver microsomes, pool of 60; pooled 1:1 male: female human liver microsomes, pool of 50).

^β^Information provided by MicroLiver Technologies Laboratory (female *Lewis* rat liver microsomes). Suspensions medium was 250 mM sucrose.

**Table 2:** Overall first order kinetic constants, k_c_ (min^−1^), of the antidepressant drugs de-methylated by liver microsomes.

Drug/microsome source	HLM	RLM-SD	RLM-W	RLM-L
VNF	0.01	0.03	0.05	0.009
O-DMV	0.025	0.009	0.02	0.007
CTM	0.007	0.02	0.07	0.007
FLX	0.01	0.02	0.06	0.04
N-FLX	0.03	0.06	0.02	0.01
MTZ	0.008	0.02	0.06	0.02
MNS	0.004	0.02	0.008	0.03

HLM- human liver microsomes; RLM- rat liver microsomes; SD- *Sprague Dawley*, W- *Wistar Han* and L- *Lewis*.

**Table 3:** Individual first order rate constants of each enantiomer, *k*_1_, *k*_2_ (min^−1^), of the antidepressant drugs de-methylated by liver microsomes.

Drug/microsome source	HLM		RLM-SD		RLM-W		RLM-L	
	*k*_1_	*k*_2_	*k*_1_	*k*_2_	*k*_1_	*k*_2_	*k*_1_	*k*_2_
O-DMV	0.03	0.02	0.009	0.008	0.02	0.018	0.009	0.005
CTM	0.008	0.005	0.016	0.014	0.09	0.05	0.007	0.007
FLX	0.004	0.004	0.03	0.02	0.08	0.06	0.05	0.03
N-FLX	0.015	0.015	0.06	0.08	0.02	0.02	0.013	0.014
MTZ	0.01	0.006	0.03	0.02	0.07	0.07	0.02	0.015
MNS	0.002	0.01	0.025	0.03	0.006	0.009	0.04	0.02

HLM- human liver microsomes; RLM- rat liver microsomes; SD- *Sprague Dawley*, W- *Wistar Han* and L- *Lewis*.

**Table 4:** Enantiomeric enrichment factors, ε_ER_ (%), of the antidepressant drugs de-methylated by liver microsomes.

Drug/microsome source	HLM	RLM-SD	RLM-W	RLM-L
VNF	NE	NE	NE	NE
O-DMV	−7 ± 1 (0.98)	−9 ± 1 (0.96)	−4 ± 1 (0.91)	−59 ± 2 (0.94)
CTM	−36 ± 3 (0.95)	−10 ± 4 (0.92)	−53 ± 3 (0.95)	NE
FLX	NE	−59 ± 7 (0.99)	−54 ± 4 (0.93)	−50 ± 5 (0.96)
N-FLX	NE	−24 ± 13 (0.93)	−8 ± 1.5 (0.95)	−9 ± 1 (0.98)
MTZ	−44 ± 2 (0.98)	−31 ± 7 (0.97)	−45 ± 3 (0.95)	−23 ± 1.5 (0.94)
MNS	−237 ± 29 (0.92)	−11 ± 6 (0.92)	−38 ± 5 (0.92)	−53 ± 12 (0.98)

Data are expressed as the mean  ±  the 95% confidence interval of the slope of the regression line in the Rayleigh plots, n = 3, p < 0.05. the regression correlation coefficient, R^2^ is given in brackets. NE- no enrichment. HLM- human liver microsomes; RLM- rat liver microsomes; SD- *Sprague Dawley*, W- *Wistar Han* and L- *Lewis*.
