# Supplementary material for: Applicability of the Rayleigh equation for enantioselective metabolism of chiral xenobiotics by microsomes, hepatocytes and in-vivo retention in rabbit tissues
Source: Sci Rep. 2016 Mar 29;6:23715. doi: 10.1038/srep23715 (PMC4810358; doi:10.1038/srep23715)
Supplement: Supplementary Information [file srep23715-s1.pdf]

Supplementary

Applicability of the Rayleigh equation for enantioselective metabolism of chiral xenobiotics by microsomes, hepatocytes and in-vivo retention in rabbit tissues

Shifra Jammer[a], Faina Gelman[b*] and Ovadia Lev[a*]

[a]The Casali Center of Applied Chemistry, The Institute of Chemistry, The Hebrew University of Jerusalem, Jerusalem 91904, Israel; [b]The Geological Survey of Israel, Jerusalem 95501 , Israel.

Content:

Summary of the definitions of the fundamental Rayleigh equations S1

Overall and individual first order kinetics plots for the enantioselective biodegradation of antidepressant drugs using liver microsomes. S3- S4

Summary of the definitions of the fundamental Rayleigh equations (equation 1) (see reference 1):

(S1) $A_1 + A_2 = C$

A_1 and A_2 are the concentrations of two enantiomers.

(S2) $ln\frac{A_{i,t}}{A_{i,0}} = -k_i \times t$

A_i is A_1,A_2 or C and the subscript "$_0$"denotes initial conditions. As a convention we assign the more reactive enantiomer as A2, i.e.

(S3) $k_1 < k_2$

k_1 and k_2 are the individual first order rate constants of each enantiomer..

(S4) $ln\frac{A_{1,t}/A_{2,t}}{A_{1,0}/A_{2,0}} = -(k_1 - k_2)t = \overline{k} \times t$

(S5) $ER = A_1/A_2$

(S6) $ln\frac{ER_t}{ER_0} = \overline{k} \times t$

Expressing t as a function of C or $A_{1,t}/A_{1,0}$ or $A_{2,t}/A_{2,0}$ by eq S2 and introducing it to eq S6 leads to equations S7,S8 and S9:

(S7) $ln\frac{ER_t}{ER_0} = \varepsilon_{ER} \times ln\frac{C_t}{C_0} = -\frac{\bar{k}}{k_c} \times ln\frac{C_t}{C_0}$

(S8) $ln\frac{ER_t}{ER_0} = \varepsilon_1 \times ln\frac{A_{1,t}}{A_{1,0}} = \varepsilon_1 \times ln\frac{C_t\,(1+{}^1/_{ER_0})}{C_0(1+{}^1/_{ER_t})} = -\frac{\bar{k}}{k_1} \times ln\frac{A_{1,t}}{A_{1,0}}$

(S9) $ln\frac{ER_t}{ER_0} = \varepsilon_2 \times ln\frac{A_{2,t}}{A_{2,0}} = \varepsilon_2 \times ln\frac{C_t(1+ER_0)}{C_0(1+ER_t)} = -\frac{\bar{k}}{k_2} \times ln\frac{A_{2},t}{A_{2.0}}$

Thus, there are three ways to define the Rayleigh enrichment factors and they can be easily calculated based on one of them if the first order kinetics is known.

Figure S1. Overall and individual first order kinetics plots for the enantioselective biodegradation of antidepressant drugs using liver microsomes.

Drug/ microsome source	HLM	RLM-SD	RLM-W	RLM-L
**CTM**	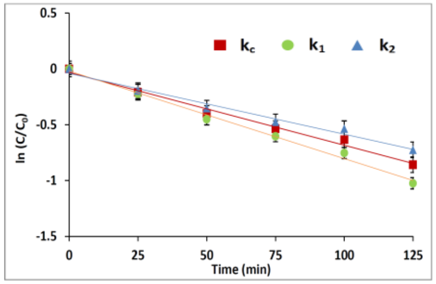	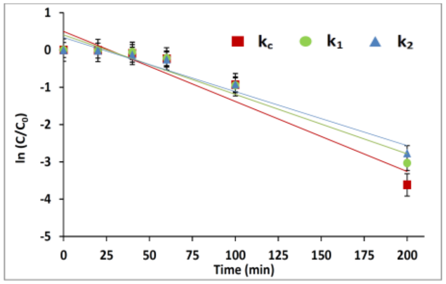	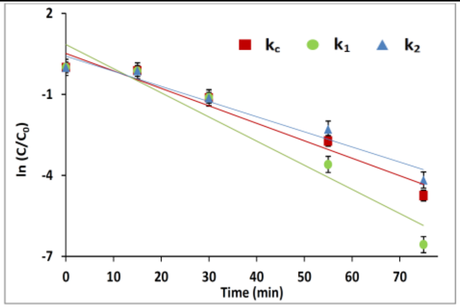	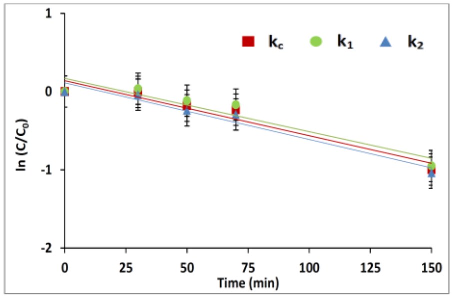
**MTZ**	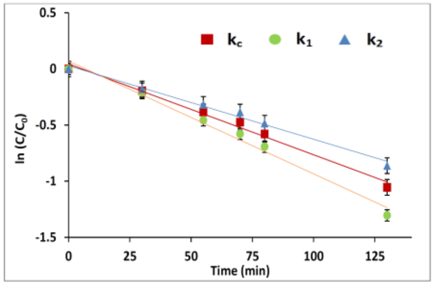	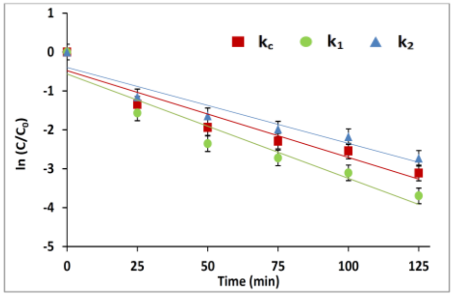	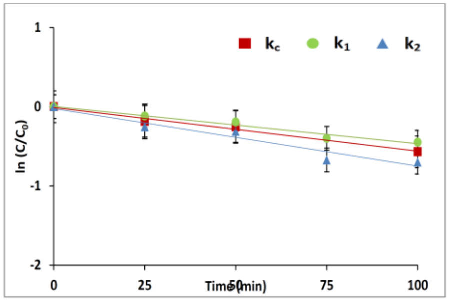	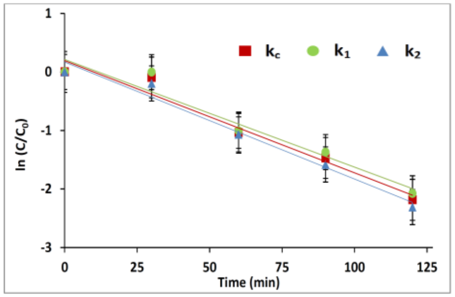
**MNS**	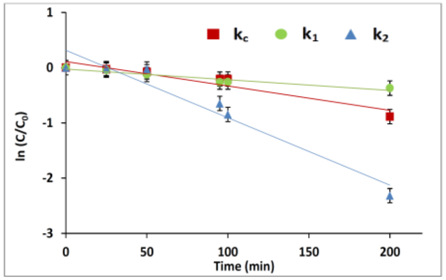	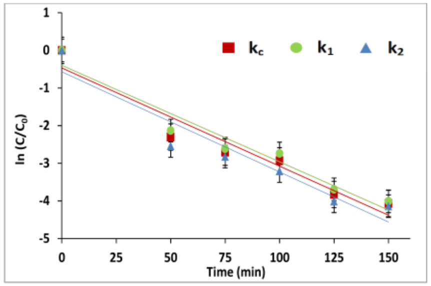	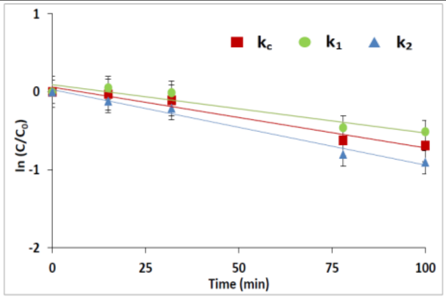	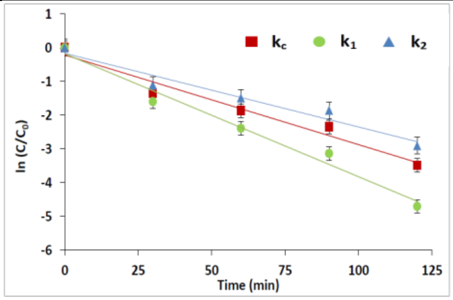

FLX

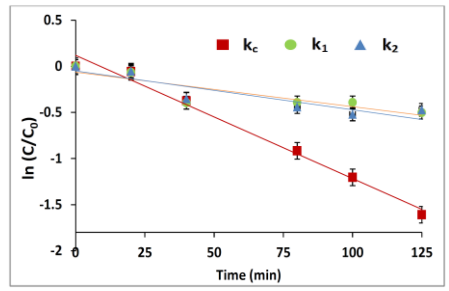

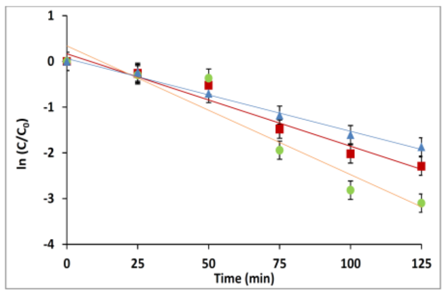

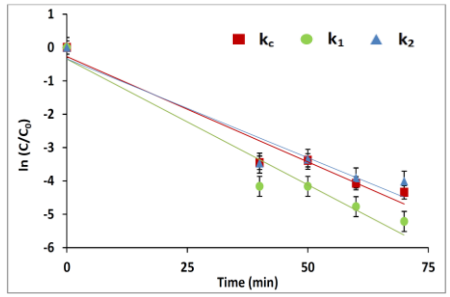

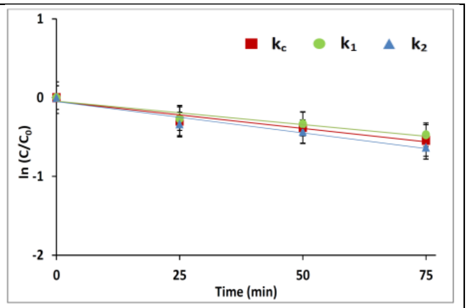

N-FLX

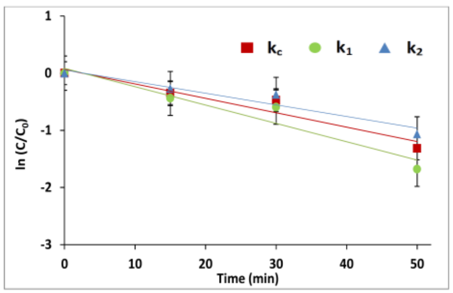

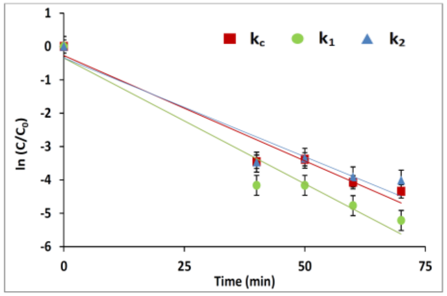

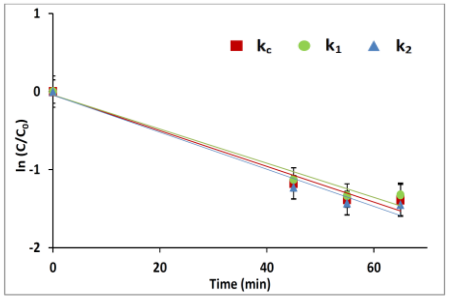

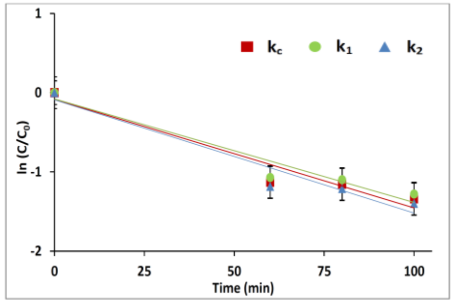

VNF

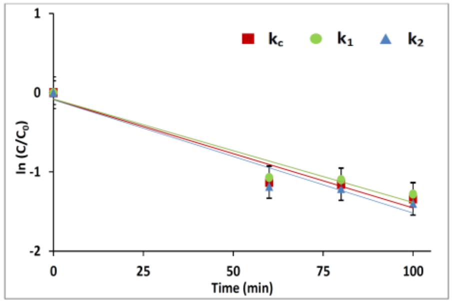

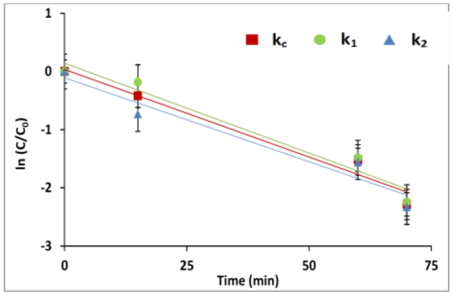

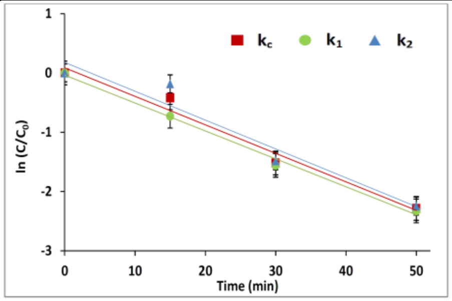

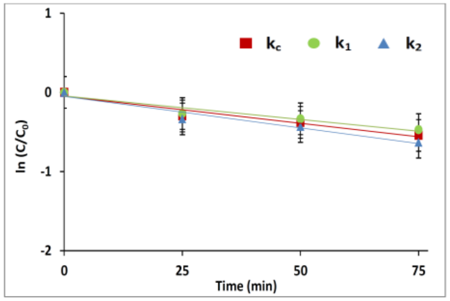

O-DMV

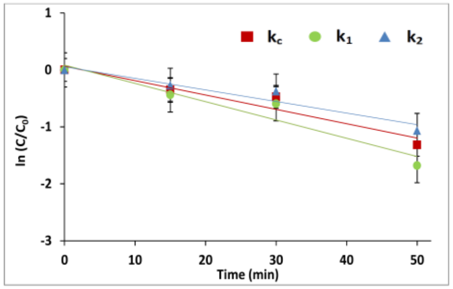

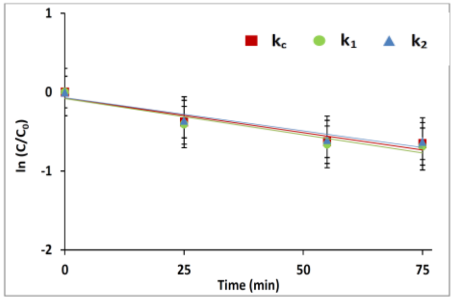

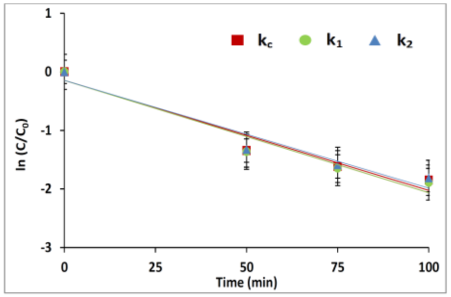

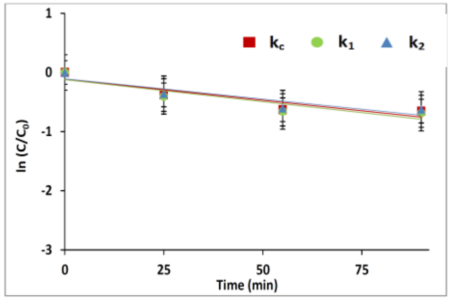

CTM-(*R*,*S*)-citalopram; MTZ -(*R*,*S*)-mirtazapine; MNS-(*R*,*S*)-mianserin; FLX-(*R*,*S*)-fluoxetine; N-FLX- (*R*,*S*)-norfluoxetine, VNF-(*R*,*S*)-venlafaxine; O-DMV-(*R*,*S*)-O-desmethylvanlafaxine. HLM- human liver microsomes; RLM- rat liver microsomes; SD-*Sprague Dawley* , W-*Wistar Han* and L-*Lewis*
